# Measurement data on the window opening behavior and climate in a strongly daylit office building

**DOI:** 10.1016/j.dib.2022.108794

**Published:** 2022-11-29

**Authors:** Sascha Hammes, Johannes Weninger

**Affiliations:** a Unit of Energy Efficient Building, University of Innsbruck, 6020 Innsbruck, Austria; b Bartenbach GmbH, research & development, 6071 Aldrans, Austria

**Keywords:** Field measurements, Indoor environment, Daylight, User behavior, Predictive models, Office buildings, Causal links

## Abstract

The long-term measurement data presented in this article result were collected in a strongly daylit office building under real working conditions and include temperature and wind speed of the outdoor situation as well as climatic variables of the indoor space, such as temperature and relative humidity. In addition to the measurement of environmental variables, the window opening behavior was also logged. The entire data acquisition was implemented via the building control system and was performed with a one-minute resolution. An exception to this is the recording of the window openings, which were logged on change of state. The measurement data obtained can be combined with other measurement data to provide an improved data basis for energy building simulations, prediction models and energy potential assessments.

**Specifications Table**

**Table:** 

Subject	Architecture
Specific subject area	Energy and environment of strongly daylit buildings
Type of data	.csv data
How the data were acquired	The field measurement of temperature and relative humidity inside (Thermokon, SR04 CO2; logged as integer value), temperature and wind speed outside (Thermokon, AGS54+; WAREMA Renkhoff SE, MWG Wind Type 3H; logged as floating-point number) and window openings (Thermokon, SRW01; logged as boolean) was carried out via a BECKHOFF Embedded PC (CX5140-0141). BECKHOFF's own TwinCAT 2 software was used to convert the measurement data collected by the sensors into a machine-precessable data format (.csv data).
Data format	Raw data
Description of data collection	The current value of temperature (inside and outside) and relative humidity as well as wind speed were recorded every minute. Logging of window openings was performed for change of state. The measurement period covers exactly 2 years (Sep 2020 to Sep 2022), except for wind measurements. Due to a gradual implementation of extensions of the data logging via the central building control system, the recording of wind measurement started later, with July 2021.
Data source location	Institution: Bartenbach GmbH Town/Region: Aldrans, Tyrol Country: Austria Latitude and longitude (and GPS coordinates, if possible) for collected samples/data: 47.24456, 11.45831
Data accessibility	Repository name: Mendeley Data Title of dataset: Measurement data on the window opening behavior and climate in a strongly daylit office building Data identification number: 10.17632/jvg8dmm3xp.1 Direct URL to data: https://data.mendeley.com/datasets/jvg8dmm3xp

## Value of the Data

• User preferences and user behavior can be significantly responsible for deviations between predicted values from building simulation and measured values from operation [1]. High-resolution measurement data collected under real conditions, such as these datasets, can be useful for researchers to develop improved simulation models, which can then help reduce the performance gaps. In this regard, the present datasets can be used specifically to test such models.
• The dataset in this article [2] also provides researchers with valuable information about the real indoor climate in a daylit open-plan office, for example, to assess the thermal comfort preferences of occupants and to perform analyses of the causal relationships between indoor climatic conditions and window opening behavior. These datasets also provide information on thermal impacts in buildings with large window areas, for example to define retrofit strategies.
• Researchers in the fields of building simulation and building controls could use these data in combination with other building measurement data to develop more accurate predictive models or predictive controls, and to compare the data with other studies conducted in other climates and application contexts.
• The datasets in this article can also help researchers in the field of building technology and control system developers to identify potential energy savings and derive measures to improve user comfort based on correlation analysis of the collected data. Control strategies could consider the user's interaction pattern with the window in the context of the historical evolution of the indoor environment (up to the user's action) from an energy perspective.
• Further, the data sets provided can help architects and building engineers to relate the energy, visual and non-visual benefits of a high daylight office building to the indoor climate. Based on such an idea, building services systems such as façade systems, HVAC and artificial lighting can be better selected and sized in the construction planning phase, as well as variants of the building envelope.

## Objective

The office building of the research and development department of Bartenbach GmbH was designed as a Living Lab. Numerous sensors record the building situation during operation in order to evaluate and further develop building controls and concepts via post-occupancy evaluations. The focus of the Living Lab is primarily on daylight and artificial light. The window opening behavior and the climate are recorded additively by sensors and made available to the scientific community as part of this Data in Brief. The measurement data can be obtained from [2].

## 1 Data Description

The measurement data are divided into four datasets. The following table (Table 1) lists the associated measured variables, including unit and measurement period:

**Table 1:** Presentation of the individual datasets with the associated measured variables, measurement periods and data points.

Dataset	Measured variable	Unit	Measuring period	Measurement rate	Number of sensors × data points
(1)	Temperature, indoor (4 sensor points)	°C	Sep 02, 2020 – Aug 31, 2022	Per minute	4 × 856,827
	Relative humidity, indoor (4 sensor points)	%	Sep 02, 2020 – Aug 31, 2022	Per minute	4 × 856,827

(2)	Temperature, outdoor	°C	Sep 02, 2020 – Aug 31, 2022	Per minute	1 × 852,688

(3)	Wind speed measured on the building roof	km/h	July 01, 2021 – Aug 31, 2022	Per minute	1 × 566,176

(4)	Window openings	Bool	Sep 02, 2020 – Aug 31, 2022	On change of state	20 × 4,574

In the datasets (1) to (3), the measurements are listed as a continuous time series in a resolution per minute and with an associated timestamp (Data were logged 24h a day). The window openings in (4) are specified as a Boolean quantity (1=window open, 0=window closed) and with reference to the time of a change of state (on open to closed and vice versa). The duration of open or closed windows can accordingly be determined from the difference of the time stamps with the respective state. All timestamps are in ISO 8601 format “YYYY-MM-DD hh:mm:ss” [3].

**Fig. 1:**
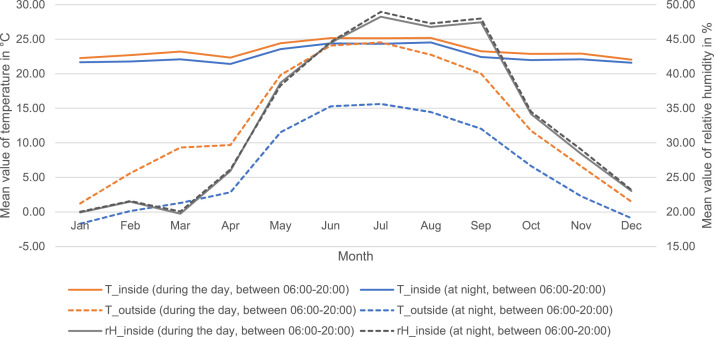
Overview of monthly mean temperatures (indoor and outdoor) and mean relative humidity over the course of the year for both daytime (06:00-20:00) and nighttime (20:00-06:00) periods.

For the recorded temperature values and for the values of relative humidity, the minima and maxima as well as mean values were determined by calendar month (Table 2) and visualized in the course of the year (Fig. 1). The absolutely measured low and high values of the indoor temperature were 17.75°C – 29.75°C (determined from the mean of all sensor points in the indoor space, minimum: Apr 06, 2021 06:46, maximum: Aug 15, 2021 16:04). For indoor relative humidity, the minima and maxima were 10.25% – 69.25% (determined from the mean of all indoor sensor points, minimum: Dec 28, 2020 01:07, maximum: July 18, 2021 14:26). In the outdoor area, the highest temperature value was recorded on Aug 15, 2021 15:44 with 44.9°C (measured on the roof) and the coldest value on Feb 14, 2021 06:51 with -14.54°C.

**Table 2:** Overview of the minimum and maximum values as well as the average values separated by day and night of indoor temperature, outdoor temperature and relative humidity in the office building of Bartenbach GmbH (location Aldrans, Austria); representation per calendar month. For the minimum and maximum values, the first detection is listed (multiple values possible). Daytime period: 06:00-20:00, nighttime period 20:00-06:00, based on the period of typical space use of the study object (see [4]).

Month	Temperature, inside (averaged over all sensors)	Relative humidity, inside (averaged over all sensors)	Temperature, outside
Jan.	Min: 19.75°C, Jan 24, 2021 05:22 Max: 26.75°C, Jan 30, 2022 15:45 Avg. ± SD, day: 22.26°C ± 1.08 Avg. ± SD, night: 21.65°C ± 0.63	Min: 11.5%, Jan 11, 2021 14:18 Max: 34.75%, Jan 08, 2022 12:21 Avg. ± SD, day: 19.91% ± 3.02 Avg. ± SD, night: 20.04% ± 2.64	Min: -12.93°C, Jan 11, 2021 08:18 Max: 18.18°C, Jan 01, 2022 12:20 Avg. ± SD, day: 1.24°C ± 5.4 Avg. ± SD, night: -1.7°C ± 4.11

Feb.	Min: 18.5°C, Feb 20, 2022 06:12 Max: 26.25°C, Feb 28, 2021 15:57 Avg. ± SD, day: 22.68°C ± 1.44 Avg. ± SD, night: 21.76°C ± 0.87	Min: 11.75%, Feb 14, 2021 12:46 Max: 34.75%, Feb 16, 2022 18:16 Avg. ± SD, day: 21.48% ± 3.78 Avg. ± SD, night: 21.58% ± 3.44	Min: -14.54°C, Feb 14, 2021 06:51 Max: 25.97°C, Feb 22, 2021 15:26 Avg. ± SD, day: 5.61°C ± 7.32 Avg. ± SD, night: 0.14°C ± 4.77

Mar.	Min: 18.5°C, Mar 28, 2021 06:22 Max: 26.25°C, Mar 13, 2022 14:28 Avg. ± SD, day: 23.2°C ± 1.33 Avg. ± SD, night: 22.09°C ± 0.93	Min: 10.75%, Mar 13, 2022 17:31 Max: 34.25%, Mar 17, 2022 16:50 Avg. ± SD, day: 19.75% ± 3.68 Avg. ± SD, night: 20.07% ± 3.45	Min: -8.44°C, Mar 21, 2021 01:01 Max: 30.9°C, Mar 31, 2021 17:03 Avg. ± SD, day: 9.33°C ± 7.63 Avg. ± SD, night: 1.3°C ± 3.96

Apr.	Min: 17.75°C, Apr 06, 2021 06:46 Max: 25°C, Apr 06, 2022 14:28 Avg. ± SD, day: 22.34°C ± 1.7 Avg. ± SD, night: 21.42°C + 1.4	Min: 18.75%, Apr 10, 2022 19:35 Max: 37.75%, Apr 01, 2021 23:26 Avg. ± SD, day: 25.92% ± 3.77 Avg. ± SD, night: 26.17% ± 4.05	Min: -6.17°C, Apr 06, 2021 06:19 Max: 30.66°C, Apr 01, 2021 15:11 Avg. ± SD, day: 9.7°C ± 7.22 Avg. ± SD, night: 2.85°C ± 4.16

May	Min: 20.75°C, May 31, 2022 03:08 Max: 28.25°C, May 20, 2022 16:35 Avg. ± SD, day: 24.41°C ± 1.6 Avg. ± SD, night: 23.56°C + 1.34	Min: 26%, May 18, 2022 13:45 Max: 50.75%, May 13, 2022 09:49 Avg. ± SD, day: 38.7% ± 4.99 Avg. ± SD, night: 38.32% ± 4.14	Min: 3.97°C, May 03, 2022 05:34 Max: 39.94°C, May 20, 2022 14:57 Avg. ± SD, day: 19.81°C ± 7.66 Avg. ± SD, night: 11.56°C ± 3.73

June	Min: 21.75°C, June 30, 2021 23:46 Max: 29.75°C, June 19, 2022 17:25 Avg. ± SD, day: 25.16°C ± 1.71 Avg. ± SD, night: 24.38°C ± 1.46	Min: 28.75%, June 24, 2021 14:59 Max: 60.5%, June 29, 2021 09:26 Avg. ± SD, day: 44.49% ± 4.79 Avg. ± SD, night: 44.59% ± 3.75	Min: 8.36°C, June 11, 2022 05:50 Max: 43.43°C, June 19, 2022 13:11 Avg. ± SD, day: 24.07°C ± 7.98 Avg. ± SD, night: 15.28°C ± 4.41

July	Min: 19.75°C, July 10, 2021 06:48 Max: 29°C, July 22, 2022 17:23 Avg. ± SD, day: 25.12°C ± 2.01 Avg. ± SD, night: 24.32 ± 1.99	Min: 33.5%, July 06, 2022 14:11 Max: 69.25%, July 18, 2021 14:26 Avg. ± SD, day: 48.27% ± 6.25 Avg. ± SD, night: 48.97% ± 6.10	Min: 9.51°C, July 10, 2021 05:32 Max: 44.14°C, July 20, 2022 15:01 Avg. ± SD, day: 24.55°C ± 7.31 Avg. ± SD, night: 15.63°C ± 3.22

Aug.	Min: 19.75°C, Aug 02, 2021 05:00 Max: 29.75°C, Aug 15, 2021 16:04 Avg. ± SD, day: 25. 2°C ± 1.67 Avg. ± SD, night: 24.54°C ± 1.39	Min: 33.5%, Aug 23, 2022 16:50 Max: 60.75%, Aug 01, 2021 08:27 Avg. ± SD, day: 46.76% ± 4.52 Avg. ± SD, night: 47.29% ± 4.28	Min: 8.14°C, Aug 29, 2021 05:58 Max: 44.9°C, Aug 15, 2021 15:44 Avg. ± SD, day: 22.76°C ± 7.66 Avg. ± SD, night: 14.47°C ± 3.33

Sep.	Min: 18.25°C, Sep 27, 2020 07:03 Max: 26.75°C, Sep 12, 2021 16:18 Avg. ± SD, day: 23.24°C ± 1.75 Avg. ± SD, night: 22.41°C ± 1.48	Min: 30.75%, Sep 08, 2021 16:29 Max: 59.5%, Sep 10, 2020 10:00 Avg. ± SD, day: 47.43% ± 4.93 Avg. ± SD, night: 48.01% ± 4.96	Min: 0.06°C, Sep 26, 2020 00:05 Max: 37.6°C, Sep 19, 2020 15:19 Avg. ± SD, day: 20.02°C ± 7.84 Avg. ± SD, night: 12.03°C ± 3.67

Oct.	Min: 19.75°C, Oct 11, 2021 06:27 Max: 27.25°C, Oct 24, 2021 15:52 Avg. ± SD, day: 22.88°C ± 1.3 Avg. ± SD, night: 21.96°C ± 0.9	Min: 10.25%, Oct 30, 2021 13:14 Max: 51.25%, Oct 05, 2021 11:51 Avg. ± SD, day: 34.17% ± 5.8 Avg. ± SD, night: 34.5% ± 5.42	Min: -1.97°C, Oct 14, 2020 07:24 Max: 29.44°C, Oct 20, 2021 14:21 Avg. ± SD, day: 11.75°C ± 6.43 Avg. ± SD, night: 6.64°C ± 4.38

Nov.	Min: 20.5°C, Nov 01, 2020 02:45 Max: 27.5°C, Nov 21, 2021 14:33 Avg. ± SD, day: 22.92°C ± 1.14 Avg. ± SD, night: 22.09°C ± 0.62	Min: 19.25%, Nov 25, 2020 12:36 Max: 45.25%, Nov 03, 2020 17:46 Avg. ± SD, day: 28.39% ± 4.62 Avg. ± SD, night: 29.02% ± 4.59	Min: -5.78°C, Nov 30, 2020 23:29 Max: 29.68°C, Nov 02, 2020 14:10 Avg. ± SD, day: 6.6°C ± 6.22 Avg. ± SD, night: 2.26°C ± 3.42

Dec.	Min: 19.75°C, Dec 18, 2021 06:20 Max: 26.5°C, Dec 31, 2021 14:54 Avg. ± SD, day: 22.04°C ± 0.94 Avg. ± SD, night: 21.59°C ± 0.58	Min: 10.25%, Dec 28, 2020 01:07 Max: 34.75%, Dec 09, 2021 20:19 Avg. ± SD, day: 23.04% ± 2.92 Avg. ± SD, night: 23.21% ± 2.97	Min: -10.93°C, Dec 22, 2021 06:51 Max: 17.4°C, Dec 31, 2021 13:03 Avg. ± SD, day: 1.51°C ± 4.29 Avg. ± SD, night: -0.9°C ± 3.18

Foehn winds occur regularly in the Alpine region of Tyrol. The external screen on the south façade, which is used not only for glare protection but also to prevent overheating, retracts at wind speeds greater than 65 km/h to protect the unit. In these cases, there is accordingly an increased thermal input, which must be compensated for by ventilation. In terms of wind speed, the measured maximum peak value was 92.14 km/h on Oct 30, 2021. Values above 65 km/h occurred on 8 of 406 measurement days.

**Fig. 2:**
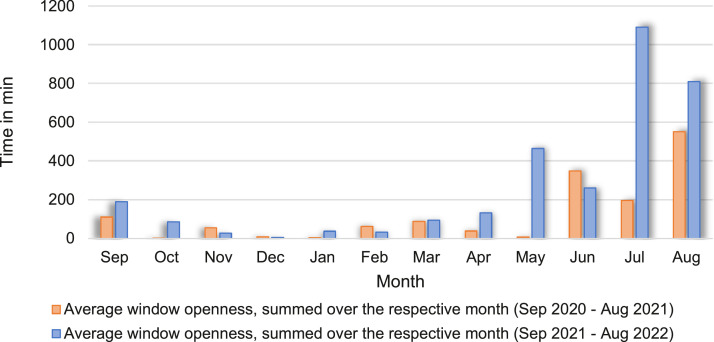
Time of open window summed over a calendar month, averaged over the 20 window contacts of the open-plan office, divided into two periods: (1) Sep. 2020 to Aug. 2021 and (2) Sep. 2021 to Aug. 2022.

The open window periods summed over a calendar month and averaged over the 20 window contacts of the open-plan office are shown in Fig. 2. A differentiation is made between the first year of the entire measurement period (Sep 2020 to Aug 2021) and the second year (Sep 2021 to Aug 2022).

## 2 Experimental Design, Materials and Methods

### 2.1 Building specifications and location information

**Fig. 3:**
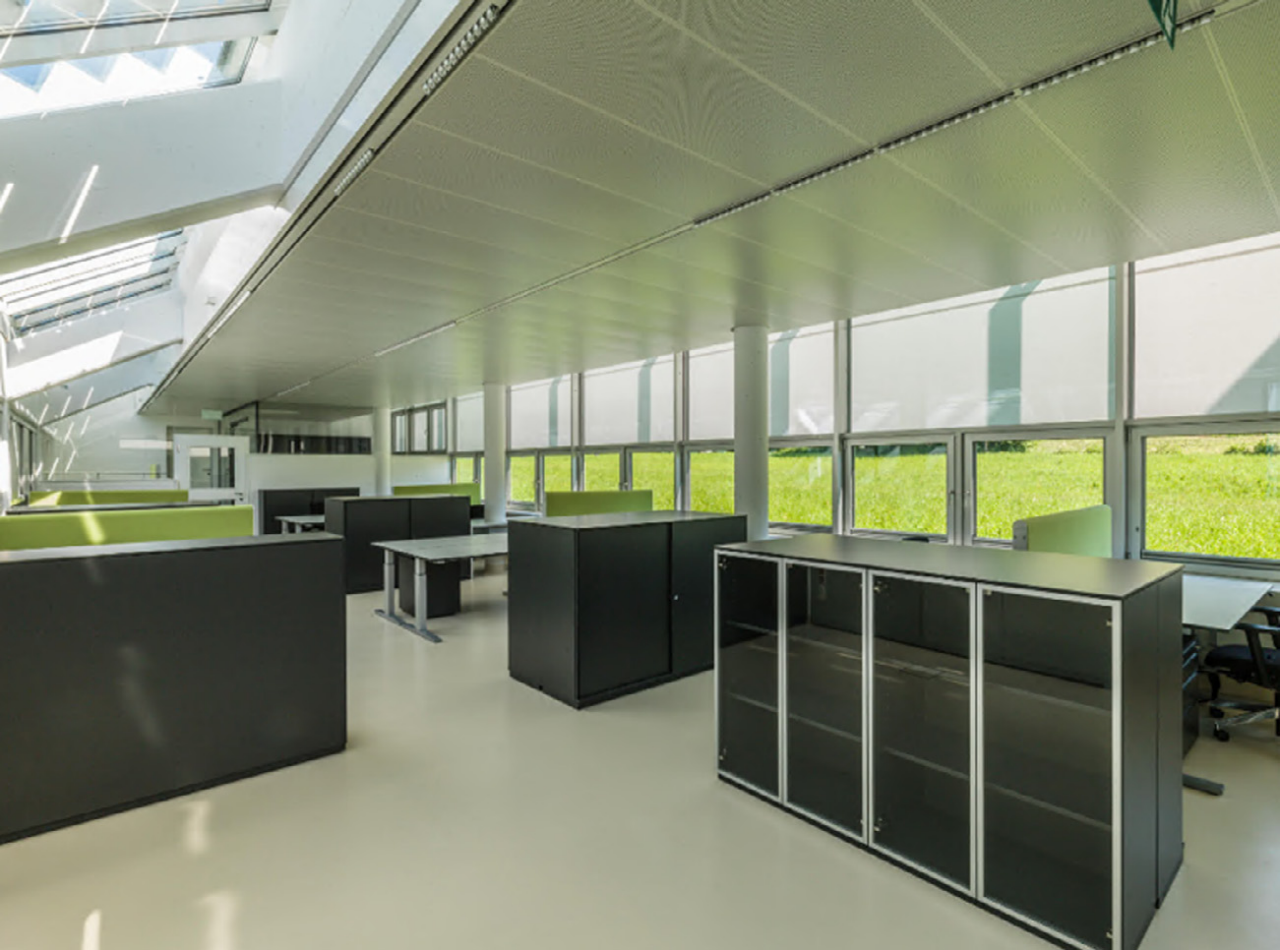
View through the open-plan office towards south-east (cf. marking of building section B in the floor plan, Fig. 4), on the right is the glazed south facade, the smaller lower windows can be opened manually and their position (open/closed) is detected by sensors (sensor position marked in Figs. 4 and 5), for the larger windows at the top the external screen is closed in the photo. Image source: Bartenbach GmbH.

The Bartenbach GmbH office building (Fig. 3), located in Aldrans, Austria, which serves as the study object, is situated in a humid-cool temperate zone according to the descriptive climate classification. According to the Köppen-Geiger climate classification, the target area is subject to a humid summer warm continental climate (Dfb) [5,6]. In addition, the Austrian Central Institute for Meteorology and Geodynamics (ZAMG) provides online data on air temperature, precipitation and sunshine duration for the nearby city of Innsbruck in a monthly, seasonal and annual representation [7].

**Fig. 4:**
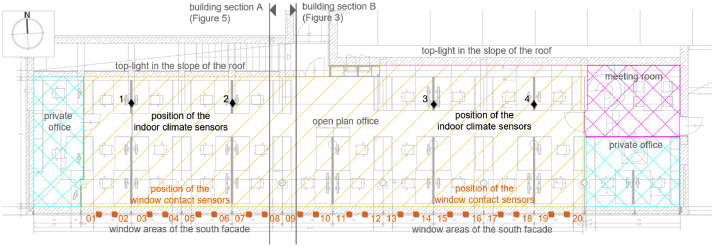
Floor plan of the Bartenbach GmbH office building showing the individual functional rooms (hatched) and sensors in the interior (orange: window contact sensors; black: sensors for recording temperature and relative humidity). Image source: Bartenbach GmbH.

**Fig. 5:**
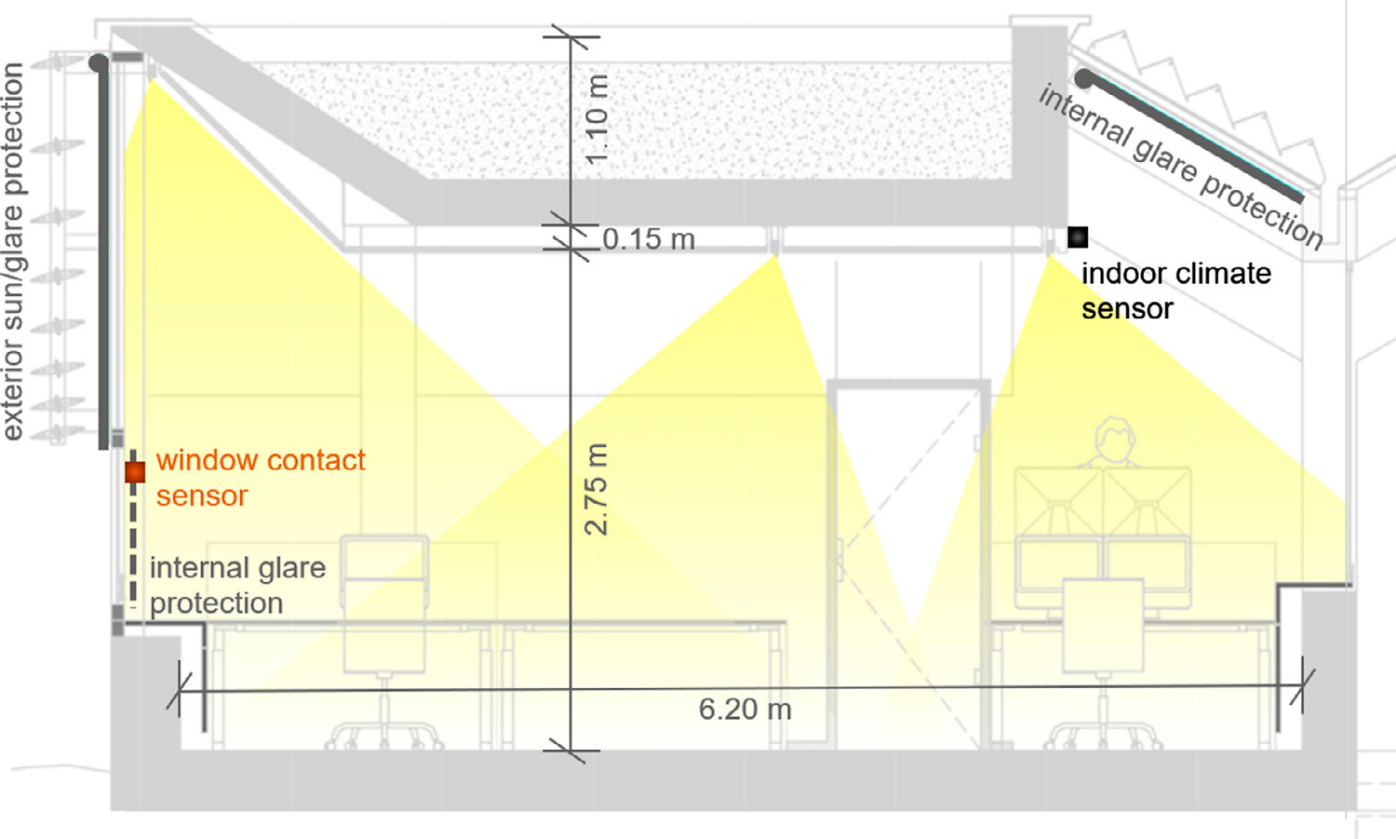
Building section A (see also Fig. 4) of the Bartenbach open-plan office, including the positions of the sensors in the interior. Image source: Bartenbach GmbH.

The multifunctional office building has one floor and a basement. The office space consists of an open-plan office (161.7 m², 28 workplaces), in which the temperature and relative humidity were measured, and two individual offices separated by transparent glass walls (15.6 m² and 14.7 m²), as well as a meeting room (9.7 m²) – see Fig. 4. A south façade with large window areas and a north-facing skylight can create a high level of daylight autonomy (daylight autonomy: DA_500;8-18_ = 81.56% (based on 500 lx according to EN12464-1, 08:00-18:00, excluding daylight saving time, including logic to avoid glare; further details on the daylight simulation in [8]). To avoid associated glare, there are external static daylight louvres, specially optimized to the geographical location of the building, as well as internal and external automatically controlled screens (Fig. 5). The ceiling height in the center of the room is 2.75 m (bottom of suspended ceiling; 2.90 m bottom of raw ceiling) and rises along the south façade to a room height of almost 4.00 m (bottom of raw ceiling) - see Fig. 5. The glazed south façade consists of 13 elements with three windows each (total window element: width x height = approx. 2.40 m x 3.20 m). The walls consist of approximately 30 cm thick reinforced concrete. Details of the lighting and control concept of the study object are described in the studies [4] and [8]. The core working hours in this office building are Monday to Thursday from 09:00-12:00 and 14:00-17:00 and Fridays from 09:00-12:00.

### 2.2 Measurement methodology

**Fig. 6:**
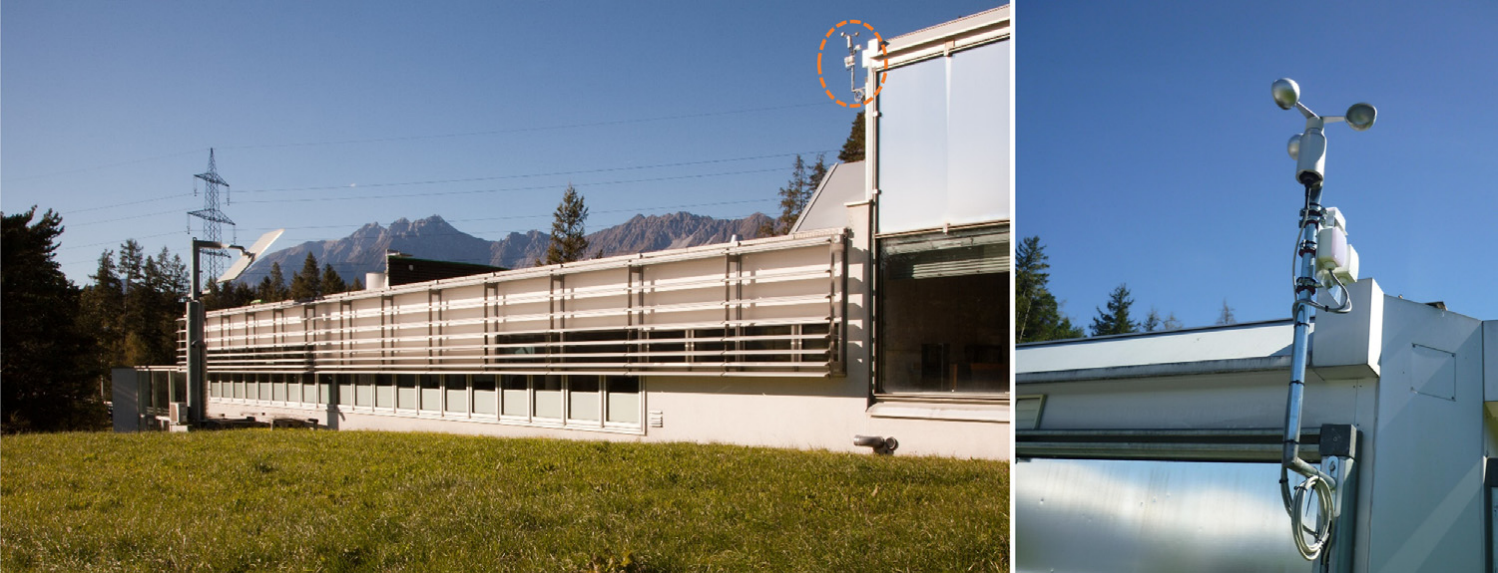
Exterior situation of the Bartenbach building with a view of the window areas of the south façade and the adjacent extension, on whose roof the wind monitor and temperature sensor are mounted (marked in orange on the figure). Image source: Bartenbach GmbH.

The measurement data from [2] were logged via a BECKHOFF embedded PC (CX5140-0141), on which the integral building control system is implemented. BECKHOFF's own TwinCAT 2 software was used to convert the measurement data collected by the sensors into a machine-processable data format (.csv). The measurement data of the four multi-sensors for recording temperature and relative humidity in the interior (Thermokon, SR04, CO2) were transmitted to the building control system via EnOcean protocol. The measuring range of this multi-sensor is 0°C to 51°C (accuracy: ±1%) and 0% to 100% (accuracy: ±3% between 20...80% rH). Since the CO_2_ values were not checked with a calibrated device during the measurement period, the CO_2_-Data were not attached to the Data in Brief. The status changes of the window contact sensors (Thermokon, SRW01) were also transmitted via EnOcean protocol. The measured values of the outdoor temperature sensor and wind sensor were read in as analogue signals via the building control system, converted and logged. The outdoor sensors are located on the roof of a directly adjacent extension to the office building (see Fig. 6). The measuring range of the outdoor temperature sensor (Thermokon, AGS54+) is -35°C to 90°C with an accuracy of typ. ±0.3°C. The wind sensor (WAREMA, MWG Wind Type 3H) has a measuring range of 0.3 m/s to 50 m/s with an accuracy of ±0.3 m/s to max. 1.5 m/s. A summary table of sensor names and variables measured by each sensor is provided in Table 3.

**Table 3:** Representation of the sensors and the variables measured by the individual sensors together with the unit of the measured variable.

Sensor name	Measured variable	Unit
Thermokon, SR04, CO2	Temperature, indoor and relative humidity, indoor	°C %

Thermokon, AGS54+	Temperature, outdoor	°C

WAREMA, MWG Wind Type 3H	Wind speed, measured on the building roof	km/h

Thermokon, SRW01	Window openings	Bool

### 2.3 Limitations of the data collection

In April/May 2021 and April 2022, the data logging of the temperature and humidity measurements was interrupted for several days. This did not affect the data logging of the window openings. For electrical connection reasons, the multi-sensors were installed in a higher position than the normative recommendation according to ASHRAE Standard 55:2013 [9]. In the context of the COVID-19 situation and the resulting greater awareness of the possibility of remote work, lower occupancy is expected, especially for the lockdowns that fall within the data collection period ((1): Mar 16, 2020 to Apr 30, 2020 [10]; (2): Nov 17, 2020 to Dec 06, 2020 [11,12]; (3): Dec 26, 2020 to Feb 07, 2021 [12]; (4): Nov 22, 2021 to Dec 11, 2021 [13]).

## Ethics Statements

This Data in Brief does not contain any personal data.

## CRediT authorship contribution statement

**Sascha Hammes:** Conceptualization, Methodology, Investigation, Data curation, Formal analysis, Visualization, Writing – original draft. **Johannes Weninger:** Conceptualization, Methodology, Investigation, Data curation, Formal analysis, Supervision, Project administration, Writing – review & editing.

## Declaration of Competing Interest

The authors declare that they have no known competing financial interests or personal relationships that could have appeared to influence the work reported in this paper.

## Data Availability

Measurement data on the window opening behavior and climate in a strongly daylit office building (Original data) (Mendeley Data).

## Appendix Supplementary materials

Image, application 1
